
JOURNAL INFORMATION
==============================
NLM Title Abbreviation: CPT Pharmacometrics Syst Pharmacol
Journal ID: CPT Pharmacometrics Syst Pharmacol
NLM Journal ID: 101580011
Journal ID: PSP4

CPT: Pharmacometrics & Systems Pharmacology

EISSN: 2163-8306
Publisher: Wiley

ARTICLE INFORMATION
==============================
PMCID: PMC11474571
DOI: 10.1002/psp4.12825
Article ID: PSP412825
Article version: 1
Subjects: Issue Information, Issue Information

Issue Information

Electronic publication date: 2023 Oct 18
Publication date: 2023 Oct
Volume: 12
Issue: 10
First page: 1371
Last page: 1374
Copyright: © 2023 The Authors. CPT: Pharmacometrics & Systems Pharmacology published by Wiley Periodicals LLC on behalf of American Society for Clinical Pharmacology and Therapeutics.
License: This is an open access article under the terms of the http://creativecommons.org/licenses/by/4.0/ License, which permits use, distribution and reproduction in any medium, provided the original work is properly cited.
License URL: https://creativecommons.org/licenses/by/4.0/

Figure count: 0
Table count: 0
Page count: 3

source-schema-version-number: 2.0
cover-date: October 2023
details-of-publishers-convertor: Converter:WILEY_ML3GV2_TO_JATSPMC version:6.4.9 mode:remove_FC converted:15.10.2024

==============================

An Official Journal of ASCPT and ISoP

### CPT: Pharmacometrics & Systems Pharmacology

Open Access

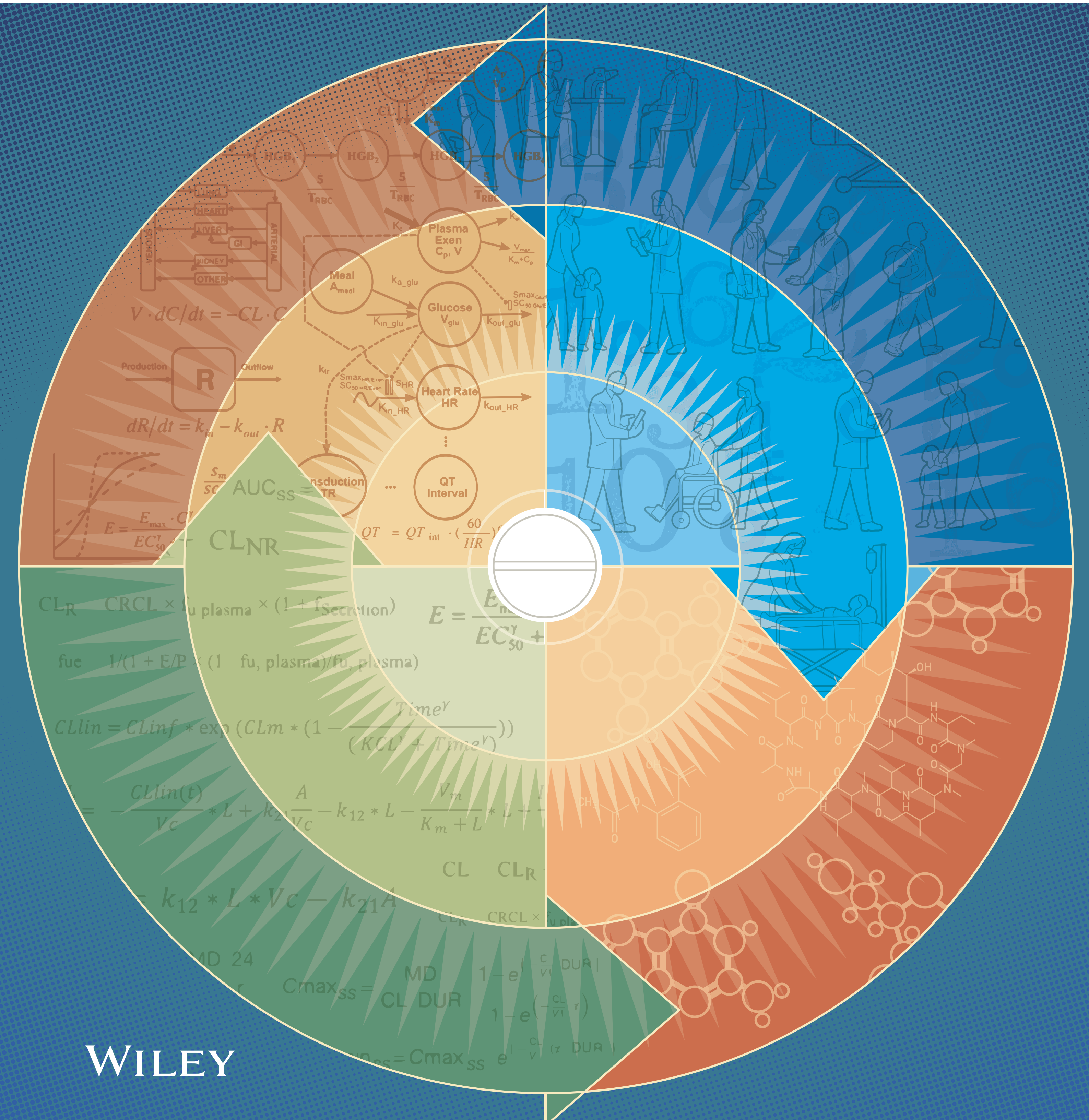

WILEY

### CPT: Pharmacometrics & Systems Pharmacology

WILEY

### Editor-in-Chief

France Mentré, MD, PhD, *School of Medicine of University Paris Diderot, Paris, France*

### Deputy Editor-in-Chief

Karen Rowland Yeo, PhD, *Certara UK Ltd., Sheffield, UK*

### Associate Editors

Paolo Denti, PhD, *University of Cape Town, Cape Town, South Africa*
Jonathan French, ScD, *Metrum Research Group, Tariffville, CT, USA*
Stefanie Hennig, MSc, PhD, *Certara Strategic Consulting, Brisbane, Australia*
Priya Jayachandran, PharmD, *Regeneron Pharmaceuticals, Inc., San Francisco, CA, USA*
Andrew Hooker, PhD, *Uppsala University, Uppsala, Sweden*
Sriram Krishnaswami, PhD, *Pfizer, Groton, CT, USA*
Ping Zhao, PhD, *Bill & Melinda Gates Foundation, Seattle, WA, USA*

### Editors-in-Training

Brian Cicali, PhD, *University of Florida, College of Pharmacy, Orlando, FL, USA*
Jane Knöchel, PhD, *AstraZeneca , Mölndal, Sweden*

### American Society for Clinical Pharmacology and Therapeutics

***Chief Executive Officer***
Abigail W. Gorman, *Alexandria, VA*
***Chief Operating Officer***
Elise S. Laffman-Johnson, *Alexandria, VA*
***Managing Editor***
Alethea B. Gerding, *Alexandria, VA*
***Deputy Managing Editor***
Alaina Webster, *Alexandria, VA*
***Senior Editorial Coordinator***
Aimee Greene, *Alexandria, VA*

### Editorial Board

Vivaswath S. Ayyar, PhD, *Janssen Research & Development, Spring House, PA*
Robert Bies, PharmD, PhD, *State University of New York at Buffalo, Buffalo, NY, USA*
Marc Birtwistle, PhD, *Clemson University Clemson, SC, USA*
Yuan Chen, PhD, *Genentech South San Francisco, CA, USA*
Marylore Chenel, PharmD, PhD, *Pharmetheus Uppsala, Sweden*
Emmanuel Chigutsa, PhD, *Eli Lilly and Company, Indianapolis, IN, USA*
Teresa Dalla Costa, PhD, *Federal University of Rio Grande do Sul - UFRGS Porto Alegre, Brazil*
Rajat Desikan, PhD, *GSK, UK, Middlesex, UK*
Narendra M Dixit, PhD, *Indian Institute of Science, Bengaluru, India*
Anne-Gaelle Dosne, PharmD, PhD, *Janssen Research & Development, Beerse, Belgium*
Thomas Dumortier, Eng, MSc, *Novartis Pharma AG, Basel, Switzerland*
James M. Gallo, PharmD, PhD, *University at Buffalo, Buffalo, NY, USA*
Wei Gao, PhD, *EMD Serono, Billerica, MA, USA*
Manish Gupta, PhD, FCP, *GenMab; Princeton, NJ, USA*
Bart Hendriks, PhD, *Novartis Institutes for BioMedical Research Cambridge, MA, USA*
Nick Holford, MBChB, *The University of Auckland Auckland, New Zealand*
Brett A. Howell, PhD, *DILIsym Services Division, Simulations Plus, Inc., Research Triangle Park, NC, USA*
Manuel Ibarra, PhD, *Universidad de la República, Montevideo, Uruguay*
Siv Jönsson, PhD, *Uppsala University Sweden, Uppsala, Sweden*
Kristin Karlsson, PhD, *Uppsala University Uppsala, Sweden*
Helen Kastrissios, PhD, *QuanTx Consulting, Brisbane, Australia*
Ron Keizer, PhD, *InsightRX San Francisco, CA, USA*
Steven E. Kern, PhD, *Global Health Labs, Bellevue, WA, USA*
Akash Khandelwal PhD, *UCB Biosciences GmbH, Monheim am Rhein, Germany*
Andrzej Kierzek, PhD, *Certara QSP; Sheffield, United Kingdom*
Qi Liu, PhD, *US Food and Drug Administration, Silver Spring, MD*
James Lu, PhD, *Genentech, Inc., South San Francisco, CA, USA*
Rajanikanth (Raj) Madabushi, PhD, *US Food and Drug Administration, Silver Spring, MD*
Efthymios Manolis, MSc, *European Medicines Agency London, United Kingdom*
Samer Mouksassi, PharmD, PhD, *Certara, Cairo, Egypt*
Jackson K. Mukonzo, PhD, *Makerere University, Kampala Uganda*
Joakim Nyberg, PhD, *Pharmetheus, Uppsala, Sweden*
Elsje Pienaar, PhD, *Weldon School of Biomedical Engineering, Purdue University, West Lafayette, IN, USA*
Daniel Scholes Rosenbloom, PhD, *Merck & Co., Rahway, NJ, USA*
Joe Standing, PhD, *University College London London, United Kingdom*
Natasha Strydom, PhD, *Amgen, Inc., San Francisco, CA, USA*
Yuichi Sugiyama, PhD, *Riken Innovation Center Sugiyama Laboratory Yokohama, Japan*
Elin Svensson, PhD, *Uppsala University, Uppsala, Sweden*
Joel Tarning, PhD, *Mahidol-Oxford Tropical Medicine Research Unit Bangkok, Thailand*
Nadia Terranova, PhD, *Merck KGaA, Lausanne, Switzerland*
Michael Tortorici, PharmD, PhD, *Aro Biotherapeutics, Philadelphia, PA, USA*
Coen van Hasselt, PhD, *Leiden University, Leiden, the Netherlands*
Paolo Vicini, PhD, MBA, *Confo Therapeutics, Ghent, Belgium*
Camille Vong, PhD, *Novartis AG Pharma, Basel, Switzerland*
Xinyuan (Susie) Zhang, PhD, *Daiichi Sankyo, Inc. Basking Ridge, NJ, USA*

### Overview

*CPT: Pharmacometrics & Systems Pharmacology* (PSP) is an official open access journal of the American Society for Clinical Pharmacology and Therapeutics (ASCPT) and International Society of Pharmacometrics (ISoP).

*CPT: Pharmacometrics & Systems Pharmacology* is a cross-disciplinary journal devoted to publishing advances in quantitative (e.g., modeling and simulation) methods as applied in pharmacology, physiology and therapeutics in humans. The journal welcomes original research articles, reviews and tutorials that bridge the following areas: pharmacometrics, modeling and simulation as applied to the design and evaluation of clinical trials, systems pharmacology modeling, particularly with a mechanistic link to human (patho)physiology, disease modeling, "population" or mixed-effects pharmacokinetics and pharmacodynamics (PKPD) modeling, modeling and simulation to support translational research, physiologically-based pharmacokinetics (PBPK), model-based meta-analyses of clinical trials, mechanism-based pharmacokinetic-pharmacodynamic modeling, computational pharmacology, bioinformatics, comparative efficacy, effectiveness and cost-effectiveness. Systems pharmacology may involve the application of systems biology approaches to study drug activities, targets and effects. The discipline is often defined with reference to engineering and pharmacological principles as the quantitative analysis of the dynamic interactions between drugs and a biologic system that aims to understand the behavior of the system as a whole. The common focus will be on quantitative methods that improve our understanding of pharmacology and therapeutics in humans.

**Abstracting and Indexing**

- Embase (Elsevier)
- SCOPUS (Elsevier)
- MEDLINE/PubMed indexing

**Society Information**

The American Society for Clinical Pharmacology and Therapeutics (ASCPT) was founded in 1900, and consists of over 2,200 professionals whose primary interest is to advance the science and practice of clinical pharmacology and translational medicine for the therapeutic benefit of patients and society. ASCPT is the largest scientific and professional organization serving the disciplines of Clinical Pharmacology and Translational Medicine.

**All articles published in this journal are freely available to read, download, and share. Articles are published under the terms of the Creative Commons Attribution Non-Commercial License which permits use, distribution, and reproduction in any medium, provided the original work is properly cited and is not used for commercial purposes.**

**Disclaimer**

The Publisher, the American Society for Clinical Pharmacology and Therapeutics, the International Society of Pharmacometrics, and Editors cannot be held responsible for errors or any consequences arising from the use of information contained in this journal; the views and opinions expressed do not necessarily reflect those of the Publisher, the American Society for Clinical Pharmacology and Therapeutics, the International Society of Pharmacometrics or Editors, neither does the publication of advertisements constitute any endorsement by the Publisher, the American Society for Clinical Pharmacology and Therapeutics, the International Society of Pharmacometrics or Editors of the products advertised.

**Publisher**

*CPT: Pharmacometrics & Systems Pharmacology* is published by Wiley Periodicals, LLC, 101 Station Landing, Suite 300, Medford, MA 02155; Telephone: 781-388-8200; Fax: 781-388-8210.

CPT: Pharmacometrics & Systems Pharmacology

### TUTORIAL

### TUTORIAL

1375 Improving pharmacometrics analysis efficiency using DataCheQC: An interactive, Shiny-based app for quality control of pharmacometrics datasets
**Or Dotan, Andrijana Radivojevic and Rajendra Singh**

### RESEARCH

### ARTICLES

1386 Pharmacometrics enhanced Bayesian borrowing approach to improve clinical trial efficiency: Case of empagliflozin in type 2 diabetes
**Lucie Fayette, Martin Oliver Sailer and Alejandro Perez-Pitarch**

1398 Optimized infusion rates for N,N-dimethyltryptamine to achieve a target psychedelic intensity based on a modeling and simulation framework
**Emma Eckernäs, Jeroen Koomen, Christopher Timmermann, Robin Carhart-Harris, Daniel Röshammar and Michael Ashton**

1411 Longitudinal efficacy and safety modeling and simulation framework to aid dose selection of belantamab mafodotin for patients with multiple myeloma
**Jon Collins, Martijn van Noort, Chetan Rathi, Teun M. Post, Herbert Struemper, Roxanne C. Jewell and Geraldine Ferron-Brady**

1425 Item response theory in early phase clinical trials: Utilization of a reference model to analyze the Montgomery-Åsberg Depression Rating Scale
**Marije E. Otto, Kirsten R. Bergmann, Marieke L. de Kam, Kasper Recourt, Gabriël E. Jacobs and Michiel J. van Esdonk**

1437 Multivariate modeling of magnetic resonance biomarkers and clinical outcome measures for Duchenne muscular dystrophy clinical trials
**Sarah Kim, Rebecca J. Willcocks, Michael J. Daniels, Juan Francisco Morales, Deok Yong Yoon, William T. Triplett, Alison M. Barnard, Daniela J. Conrado, Varun Aggarwal, Ramona Belfiore-Oshan, Terina N. Martinez, Glenn A. Walter, William D. Rooney and Krista Vandenborne**

1450 SARS-CoV-2 viral dynamic modeling to inform model selection and timing and efficacy of antiviral therapy
**Shengyuan Zhang, Akosua A. Agyeman, Christoforos Hadjichrysanthou and Joseph F. Standing**

1461 Physiologically-based pharmacokinetic modeling for investigating the effect of simeprevir on concomitant drugs and an endogenous biomarker of OATP1B
**Shinji Nakayama, Kota Toshimoto, Shinji Yamazaki, Jan Snoeys and Yuichi Sugiyama**

1473 Model based assessment of food and acid reducing agent effects on oral absorption of mezigdomide (CC-92480), a novel cereblon E3 ligase modulator
**Fan Wu, Liangang Liu, Allison Gaudy, Xiaomin Wang, Leon Carayannopoulos, Michael Pourdehnad and Manisha Lamba**

1485 Mechanistic modeling projections of antibody persistence after homologous booster regimens of COVID-19 vaccine Ad26.COV2.S in humans
**Anna Dari, Philippe Jacqmin, Yuki Iwaki, Martine Neyens, Mathieu Le Gars, Jerald Sadoff, Karin Hardt, Javier Ruiz-Guiñazú and Juan José Pérez-Ruixo**

1499 Population pharmacokinetic analyses for belzutifan to inform dosing considerations and labeling
**Dhananjay D. Marathe, Petra M. Jauslin, Huub Jan Kleijn, Carolina de Miranda Silva, Anne Chain, Thomas Bateman, Peter M. Shaw, Anson K. Abraham, Eunkyung A. Kauh, Yanfang Liu, Rodolfo F. Perini, Dinesh P. de Alwis and Lokesh Jain**

1511 A dynamic model of the intestinal epithelium integrates multiple sources of preclinical data and enables clinical translation of drug-induced toxicity
**Louis Gall, Ferran Jardi, Lieve Lammens, Janet Piñero, Terezinha M. Souza, Daniela Rodrigues, Danyel G. J. Jennen, Theo M. de Kok, Luke Coyle, Seung-Wook Chung, Sofia Ferreira, Heeseung Jo, Kylie A. Beattie, Colette Kelly, Carrie A. Duckworth, D. Mark Pritchard and Carmen Pin**

1529 Evaluation of postprandial total triglycerides within the TIGG model for characterizing postprandial response of glucose, insulin, and GLP-1
**Jennifer Leohr and Maria C. Kjellsson**

1541 Population pharmacokinetics of apixaban in a real-life hospitalized population from the OptimAT study
**Frédéric Gaspar, Jean Terrier, Samantha Favre, Pauline Gosselin, Pierre Fontana, Youssef Daali, Camille Lenoir, Caroline Flora Samer, Victoria Rollason, Jean-Luc Reny, Chantal Csajka and Monia Guidi**

1553 A comprehensive evaluation in clinic and physiologically-based pharmacokinetic modeling and simulation to confirm lack of cytochrome P450–mediated drug–drug interaction potential for pomotrelvir
**Ziping Yang, Nathalie Rioux, Ludwig Vincent, Hannah M. Jones, David Cha, Andrew Plummer, David Wilfret and Brian P. Kearney**
